# How should individual participant data (IPD) from publicly funded clinical trials be shared?

**DOI:** 10.1186/s12916-015-0532-z

**Published:** 2015-12-17

**Authors:** C. Tudur Smith, C. Hopkins, M. R. Sydes, K. Woolfall, M. Clarke, G. Murray, P. Williamson

**Affiliations:** MRC North West Hub for Trials Methodology Research, Department of Biostatistics, University of Liverpool, Block F Waterhouse Building, Liverpool, L69 3GL UK; MRC Clinical Trials Unit, University College London, Aviation House, 125 Kingsway, London, WC2B 6NH UK; MRC North West Hub for Trials Methodology Research, Department of Psychological Sciences, Block B Waterhouse Building, Brownlow Street, Liverpool, L69 3GL UK; All-Ireland Hub for Trials Methodology Research, School of Medicine, Dentistry and Biomedical Sciences, Queen’s University Belfast, Health Sciences Building, 97 Lisburn Road, Belfast, BT9 7BL UK; Centre for Population Health Sciences, University of Edinburgh, Teviot Place, Edinburgh, EH8 9AG UK

**Keywords:** Data sharing, Individual participant data, IPD, Clinical trial, Publicly funded, CTU, Good practice

## Abstract

**Background:**

Individual participant data (IPD) from completed clinical trials should be responsibly shared to support efficient clinical research, generate new knowledge and bring benefit to patients. The Medical Research Council (MRC) Hubs for Trials Methodology Research (HTMR) has developed guidance to facilitate the sharing of IPD from publicly funded clinical trials.

**Methods:**

Development of the guidance was completed over four phases which included a focussed review of policy documents, a web-based survey of the UK Clinical Research Collaboration (CRC) Registered Clinical Trials Units (CTU) Network, participation of an expert committee and an open consultation with the UKCRC Registered CTU Network. The project was funded by the MRC HTMR (MR/L004933/1-R39).

**Results:**

Good practice principles include: (i) the use of a controlled access approach, using a transparent and robust system to review requests and provide secure data access; (ii) seeking consent for sharing IPD from trial participants in all future clinical trials with adequate assurance that patient privacy and confidentiality can be maintained; and (iii) establishing an approach to resource the sharing of IPD which would include support from trial funders, sponsor organisations and users of IPD. The guidance has been endorsed by Cancer Research UK, MRC Methodology Research Programme Advisory Group, Wellcome Trust and the Executive Group of the UKCRC Registered CTU Network. The National Institute for Health Research (NIHR) has confirmed it is supportive of the application of this guidance.

**Conclusions:**

Implementation of these principles will improve transparency, increase the coherent sharing of IPD from publicly funded trials, and help publicly funded trials to adhere to trial funder and journal requirements for data sharing.

**Electronic supplementary material:**

The online version of this article (doi:10.1186/s12916-015-0532-z) contains supplementary material, which is available to authorized users.

## Background

Before a clinical trial begins recruiting participants the trial should be ‘registered’ in a clinical trials registry such as ClinicalTrials.gov. This public record of completed and ongoing trials assures transparency and reduces the potential for publication bias, which is known to be a significant problem in medical research [1]. During a clinical trial data are collected about each individual participant. This may include participant characteristics (e.g. age, gender), clinical measurements (e.g. blood pressure, heart rate), medical history (e.g. history of diabetes), clinical laboratory results (e.g. white blood cell count), images (e.g. X-rays), adverse events (e.g. gastrointestinal bleeding events), clinical outcome (e.g. death), and details of randomisation and treatment received. These data are referred to as individual participant data (IPD).

At the end of a clinical trial, results are generated by summarising the IPD to evaluate the effect of interventions administered during the clinical trial. The summary results should be fully reported and published in medical journals [2] and trial registries, but utility of the IPD continues as they provide enormous potential to investigate further clinical and/or methodological questions beyond those that the trial had been originally designed to address. Several clinical trial funders and journals now require that the IPD from a clinical trial is made available on reasonable request [3–6] after completion of the trial.

There are numerous examples in the medical literature that demonstrate the value of IPD and what can be achieved through data sharing. This includes improving the reliability and robustness of comparative meta-analyses in cancer [7], cardiovascular disease [8] and epilepsy [9]; the reliable identification of subgroups of patients that benefit most from treatment [10]; aiding the development of new methodology [11]; and providing the best evidence to inform the development of clinical guidelines [12] and new clinical trials [13]. Greater access to IPD and clinical study reports has been incredibly useful to help overcome the problem of bias in the medical literature with high profile examples focussing on Tamiflu [14] and Paroxetine [15], in which more reliable and balanced information has been generated for patients and clinical practitioners.

Despite the advantages and potential usefulness, IPD is often unavailable [16–18], or may be shared but using responsive ad hoc approaches which limits discoverability, productivity and the potential preservation of valuable data sets. Failure to exploit existing data means that new data are collected unnecessarily which creates unacceptable waste in clinical research [19]. Attitudes are changing, and the pharmaceutical industry [20, 21], drug regulators [22] and the clinical trial community [23, 24] are taking steps to improve things. For the publicly funded trials sector there are examples of good practice [25] but progress towards sharing IPD from clinical trials using a cohesive and consistent approach is slow. More now needs to be done to encourage proactive sharing using common principles of good practice.

In this article we summarise the process used to develop a guidance document for publicly funded clinical trials and outline the key principles of good practice that aim to increase and improve the uptake of responsible data sharing in this key stakeholder group.

## Methods

Development of the guidance was funded by the Medical Research Council (MRC) Network of Hubs for Trials Methodology Research (HTMR). A project group including statisticians, clinical trialists, systematic reviewers and methodologists was established to develop the guidance, a process that was completed over four phases.

During phase 1 a focussed search for data sharing policy documents was conducted to identify good practices for responsible sharing of IPD. We searched ‘Google Search’ and the University of Liverpool Discover database (search terms provided in Additional file 1). We used NVivo software to assist the management and indexing of themes and sub-themes identified within and across policy documents. We created theme summary reports to assist the development of guidance.

During phase 2 a web-based survey of the UK Clinical Research Collaboration (CRC) Registered Clinical Trials Units (CTU) Network was undertaken to ascertain current data sharing activities, good practices and possible barriers to sharing IPD from the perspective of organisations coordinating publicly funded clinical trials. Full details of the survey methods and results have been published elsewhere [26]. In brief, a 47-item questionnaire was developed and conducted online using SelectSurvey.NET. Ethical approval was obtained from the University of Liverpool Research Ethics Committee and as the survey was conducted online, completion was regarded as consent to participate. A link to the survey was emailed to the Directors of 45 CTUs within the UKCRC Registered CTU Network in April 2014, with email reminders sent after 2, 4 and 6 weeks. The questionnaire took approximately 15 minutes to complete.

The project group used the information gathered from phase 1 and 2 to develop a draft guidance document summarising the principles of good practice. During phase 3 the draft guidance was circulated to a committee of 13 selected experts, with six representatives from UK publicly funded CTUs that generate clinical trial IPD or UK academic institutions with expertise in using IPD for research purposes, three from pharmaceutical companies in the UK and US, two from UK clinical trial funding bodies, and two from an independent company with knowledge in the area of sharing clinical trial IPD (Additional file 2: Appendix 4). A one-day meeting involving members of the project group and expert committee was held in London during November 2014 to discuss the guidance, and an iterative process used to update and revise the draft guidance to incorporate comments from the expert committee. During phase 4 the revised guidance was circulated to the Directors of the 45 UKCRC Registered CTU Network and a period of open consultation was used to obtain further comments which were incorporated into the final version of the guidance. The full guidance is available as Additional file 2 and from the MRC HTMR website (http://www.methodologyhubs.mrc.ac.uk/files/7114/3682/3831/Datasharingguidance2015.pdf). The key principles, repeated verbatim from the full guidance, are summarised in this article.

## Results

### Support for the guidance

The guidance has been endorsed by Cancer Research UK, MRC Methodology Research Programme Advisory Group, Wellcome Trust and the Executive Group of the UKCRC Registered CTU Network. The National Institute for Health Research (NIHR) has confirmed it is supportive of the application of this guidance.

### Summary of guidance

**Fig. 1:**
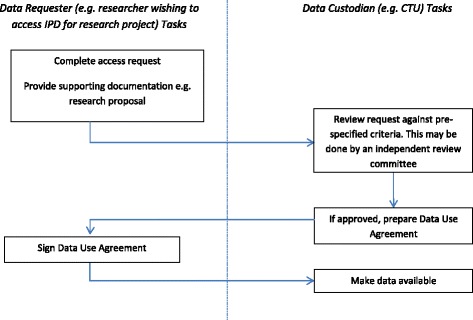
Flow chart of activities in a controlled access model

Results of the survey conducted in phase 2 have been published elsewhere [26]. In brief, the CTUs were supportive of the principle of sharing IPD but common concerns were raised about the inappropriate reuse of clinical trial data, the additional resource required for publicly funded CTUs to prepare and share data, the potential loss of ability to publish further research, and the potential risk to trial participant privacy. The use of a controlled access approach (Fig. 1) such as that used by MRC CTU at University College London (UCL) [25], the Yale University Open Data Access Project (YODA) [27] and Clinical Study Data Request (CSDR) [21] with systems in place to review data access requests from researchers, was the preferred approach and this has been assumed throughout.

**Fig. 2:**
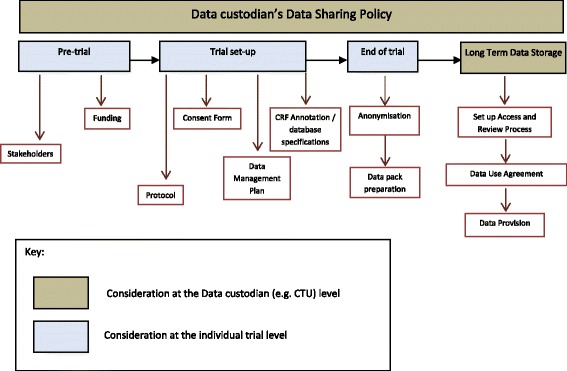
Data sharing activities through a clinical trial process

The ‘data custodian’ is defined within the guidance as a research group, company, organisation or sponsor that collects, manages and stores data from a clinical trial, and would be responsible for data sharing. The data custodian of publicly funded clinical trials would need to consider a number of data sharing activities that could arise throughout the clinical trial process (Fig. 2, Box 1 and Box 2).

Activities can be separated into those that occur at the level of a data custodian’s organisation and which are applicable across multiple trials (Box 1), and also those activities that occur each time an individual trial is conducted (Box 2). The ‘sponsor’ of the trial has ultimate responsibility for authorising the release of data but in many publicly funded clinical trials the ‘sponsor’ may delegate responsibility for activities of data collection and storage to the ‘data custodian’ (e.g. the Chief Investigator’s hosting University or NHS Trust may be the ‘sponsor’ but the ‘data custodian’ would be the CTU coordinating the trial), and so a clear process is required to ensure that sponsor approval for data sharing is provided. Further details of the principles are provided in the full guidance (Additional file 2).

## Box 1 - Good practice at the data custodian (e.g. CTU) level

*Policy*

- A data sharing policy should be developed by the data custodian organisation outlining the general approach to data sharing, summarising the elements discussed below. The policy should align with any other overarching policies, e.g. host organisation policy, funder policies

*Scope*

- IPD and associated documentation should be made available for all prospective publicly funded clinical trials. Requests for data from historical clinical trials should be dealt with on a case-by-case basis
- IPD should be made available as soon as reasonably possible, e.g. 18 months after trial completion

*Data request process*

- Sponsor approval for data sharing should be sought (sponsor might initially agree principles of data sharing but delegate responsibility for implementing data sharing to the data custodian)
- Only bona fide research groups should be eligible to access data (e.g. evidenced via CVs and the involvement of a qualified statistician)
- Data access requests should be made via an application form detailing the specific requirements and the proposed research and publication plan
- Data access requests should be reviewed against specific criteria by data custodians (e.g. trial statistician and Chief Investigator) or by an external Independent Review Panel. Decisions about requests should be made promptly according to a published schedule (no more than 3 months after receipt of request)
- Details of all data requests and their outcomes, with clear rationale for any refusals, should be made publicly available. Data requesters should be informed of this in advance

*Data release process*

- Data should be made available as soon as possible after approval of requests
- Data should be made available on a secure server or via other secure data transfer method
- Supporting documentation should be supplied with the dataset

*Data use agreement*

- A data use agreement should be utilised which, at a minimum: (i) prohibits attempts to re-identify or contact trial participants; (ii) addresses any requirements regarding planned outputs of proposed research, e.g. publication and acknowledgement requirements; and (iii) prohibits non-approved uses or further distribution of the data

*Resources*

- Funds for responsible data sharing should be requested by the original trial team from trial funders as part of initial trial grant applications, e.g. to fund dataset preparation and anonymisation
- Reasonable costs may be recovered from data requesters if appropriate but data sharing activities should not be profit generating
- Host organisations (e.g. Institute of Higher Education) may be able to provide funds for routine data sharing activities, e.g. ongoing maintenance of a data sharing system
- Responsibilities of staff for data sharing should be determined and funding should be sourced

## Box 2 - Good practice at the individual trial level

*Prior to trial funding*

- Identify data sharing stakeholders for a trial early on (e.g. sponsor, funder, Chief Investigator, trial management group, CTU) and highlight the data sharing policy
- Understand the trial funder’s policy and include plans and reasonable costs (if appropriate) for sharing IPD within the trial grant application

*During trial set-up*

- Identify roles and responsibilities for data sharing activities and include on a delegation log
- Include outline plans for data sharing in the protocol (see SPIRIT checklist item 31c [28])
- Include detailed plans for data sharing in the trial data management plan
- Include a data sharing statement in the consent form and information in the patient information leaflet. The Health Research Authority [29] currently recommend the following wording: “I understand that the information collected about me will be used to support other research in the future, and may be shared anonymously with other researchers.”
- Annotate the complete set of blank case report forms (CRFs) so that they clearly describe the data variable labels and values contained within the electronic dataset. This may not be required if dataset specifications and blank CRFs are sufficiently detailed to enable matching of data variables from CRFs to the electronic dataset (note: blank CRFs made available on the organisation’s website, or some other forum, would help researchers identify relevant data that have been collected prior to submitting a formal request for data)

*End of trial*

- Prepare the anonymised dataset ready for sharing. The level of anonymisation should be determined in conjunction with other considerations, such as original patient consent and method of data transfer
- Dataset preparation should be done by individuals with an understanding of data management and basic statistics, with quality control provided by a further individual who is independent of the process
- Prepare ‘data pack’ ready for sharing. This would typically include: (i) electronic datasets in a suitable format that is recognised by a range of statistical software, that could be easily divided to create a subset of data if required for the use case requested; and (ii) supporting documentation (minimum requirement would be protocol with amendments, blank CRFs, dataset specifications including data variable amendments). Timing of data pack preparation may be reactive or proactive.

## Conclusions and discussion

We have developed good practice guidance for organisations that conduct publicly funded clinical trials. Although the guidance has been developed with UK publicly funded trials in mind, many of the principles apply to clinical trials coordinated by the private sector and to organisations conducting clinical trials in countries other than the UK. Implementation of these principles will improve transparency and increase the coherent sharing of IPD to support clinical research and benefit patients. More research is needed to help improve the discoverability of these valuable data [30] and we would recommend that the CONSORT checklist [31] for reporting of randomised controlled trials (RCTs) be updated to include a specific item about data sharing and where IPD for the trial can be located. As many medical journals and clinical trial funders now require authors to make their data available, the wider adoption of the principles outlined in this guidance will aid compliance with funder and journal policies on data sharing.

A UKCRC data sharing task and finish group has recently been established to help encourage sharing IPD from clinical trials and support the implementation of the good practice outlined in this guidance. This is a critical component of this project and we will report on our experiences in due course. Researchers who implement this guidance are strongly encouraged to share their experience of how the principles work in practice to inform future updates of the guidance.

### Availability of data

The de-identified survey data will be made available for research purposes by contacting the first author (cat1@liv.ac.uk).

## Additional files

Additional file 1:
**Search terms used during focussed search for data sharing policy documents.** (DOCX 15 kb) Additional file 2:
**Good practice principles for sharing individual participant data from publicly funded clinical trials.** (DOCX 437 kb)
