# Supplementary material for: How should individual participant data (IPD) from publicly funded clinical trials be shared?
Source: BMC Med. 2015 Dec 17;13:298. doi: 10.1186/s12916-015-0532-z (PMC4682216; doi:10.1186/s12916-015-0532-z)
Supplement: Additional file 1: — Search terms used during focussed search for data sharing policy documents. (DOCX 15 kb) [file 12916_2015_532_MOESM1_ESM.docx]

Appendix

Search terms used during focussed search for data sharing policy documents.

1. Google search

| Clinical trial AND   - Data sharing - Data sharing policy - Data repository - Data access - Transparency - Repository - Funder policy - Publisher policy | Research AND   - Data sharing - Data sharing policy - Data repository - Data access - Transparency - Repository - Funder policy - Publisher policy |
| --- | --- |

1. University of Liverpool Discover search

| Clinical trial AND   - data sharing - transparency |
| --- |
