# Supplementary material for: How should individual participant data (IPD) from publicly funded clinical trials be shared?
Source: BMC Med. 2015 Dec 17;13:298. doi: 10.1186/s12916-015-0532-z (PMC4682216; doi:10.1186/s12916-015-0532-z)
Supplement: Additional file 2: — Good practice principles for sharing individual participant data from publicly funded clinical trials. (DOCX 437 kb) [file 12916_2015_532_MOESM2_ESM.docx]

|  |
| --- |
| GOOD PRACTICE PRINCIPLES FOR SHARING INDIVIDUAL PARTICIPANT DATA FROM PUBLICLY FUNDED CLINICAL TRIALS |
| Version 1 April 2015 |

|  |
| --- |

| 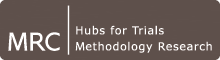 | 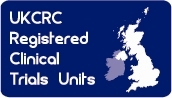 |
| --- | --- |
|  |  |
| 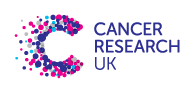 | 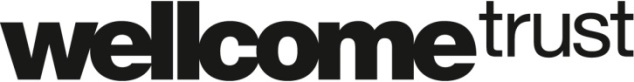 |

**Preface**

Publicly funded Clinical Trials Units (CTUs) provide infrastructure and multi-disciplinary teams of researchers that design, conduct, analyse and report clinical trials. Sharing Individual Participant Data (IPD) from clinical trials offers numerous well recognised advantages that can advance clinical research and benefit patients. The clinical trial community, including publicly funded CTUs, have a duty to facilitate this process. This document summarises good practice principles for publicly funded CTUs to follow when sharing IPD and associated documentation from a clinical trial.

Whilst there are several different approaches that could be taken to sharing IPD from clinical trials, this document focusses on a controlled access approach. This facilitates the responsible sharing of ‘richer data’ for research purposes within the confines of a system that aims to protect patient privacy. The UK Clinical Research Collaboration (UK CRC) registered CTUs unanimously supported the use of a controlled access approach to sharing IPD in a survey which informed the development of this guidance([1](#_ENREF_1)).The content of this guidance has been further informed by literature, the internet and expert opinion as part of a research project funded by the Medical Research Council (MRC) Hubs for Trials Methodology Research (MR/L004933/1-R39) led from the North West Hub at the University of Liverpool. The good practice principles proposed are the result of a consultative process and take into account several other current initiatives in the field of data sharing.

This guidance has been endorsed by Cancer Research UK, MRC Methodology Research Programme Advisory Group, Wellcome Trust and the Executive Group of the UK CRC Registered CTUs Network. The National Institute for Health Research (NIHR) has confirmed it is supportive of the application of this guidance.

Section 1 of this document provides a summary of the good practice principles for sharing IPD using a controlled access system, section 2 provides background information, section 3 discusses two data sharing models, and finally section 4 provides a detailed description of the good practice principles described in section 1.

Comments or suggestions should be directed to Dr Catrin Tudur Smith ([cat1@liv.ac.uk](mailto:cat1@liv.ac.uk)).

**Acknowledgements:**

We are particularly grateful to the following for their contributions and suggestions: Jane Armitage, Sue Bell, Jesse Berlin, Jan Bogaerts, Gill Booth, Claire Daffern, Joanne Eatock, Rob Frost, Carrol Gamble, Jamie Garner, Will Greenacre, Helen Hickey, Sally Hollis, Nazir Lone, Maike Rentel, Richard Riley, Haleema Shakur, Lesley Stewart, Liz Tremain, Peter Varnai, Neil Walker.

**Funding:**

Development of this guidance was funded by the MRC Network of Hubs for Trials Methodology Research (MR/L004933/1-R39) including support from the North West Hub, London Hub, Clinical Trial Service Unit Hub, All Ireland Hub, Midlands Hub and Edinburgh Hub.

Table of Contents

[Glossary 4](#_Toc417577073)

[1 Summary of Good Practice Principles for Sharing Individual Participant Data (IPD) from Publicly Funded Clinical Trials 5](#_Toc417577074)

[2 Background 7](#_Toc417577075)

[2.1 Aim 7](#_Toc417577076)

[2.2 Scope 7](#_Toc417577077)

[2.3 Funder requirements 7](#_Toc417577078)

[2.4 Legal requirements 8](#_Toc417577079)

[2.5 Sponsor requirements 8](#_Toc417577080)

[2.6 Regulatory requirements 8](#_Toc417577081)

[3 Data Sharing Models 9](#_Toc417577082)

[3.1 Open access 9](#_Toc417577083)

[3.2 Controlled access 9](#_Toc417577084)

[4 Good Practice Principles for Sharing Individual Participant Data (IPD) from Publicly Funded Clinical Trials 12](#_Toc417577085)

[4.1 Considerations at the CTU level - Data Sharing Policy 13](#_Toc417577086)

[4.1.1 Scope 13](#_Toc417577087)

[4.1.2 Data request process 13](#_Toc417577088)

[4.1.3 Data release process 14](#_Toc417577089)

[4.1.4 Data use agreement 14](#_Toc417577090)

[4.1.5 Resources 15](#_Toc417577091)

[4.2 Considerations at the individual trial level 16](#_Toc417577092)

[4.2.1 Prior to trial funding 16](#_Toc417577093)

[4.2.2 During trial set-up 16](#_Toc417577094)

[4.2.3 End of trial 17](#_Toc417577095)

[5 References 20](#_Toc417577096)

[Appendix 1: Examples of Documentation 21](#_Toc417577097)

[Appendix 2: List of 28 potential patient identifiers in datasets 23](#_Toc417577098)

[Appendix 3: Example Anonymisation Standard 24](#_Toc417577099)

[Appendix 4: Data Sharing Workshop Attendees 29](#_Toc417577100)

# Glossary

Throughout this document we use the following definitions:

*Anonymisation*

A process of turning data into a form which does not identify individuals and where identification is not likely to take place ([2](#_ENREF_2)).

*Clinical Trial*

Any research study that prospectively assigns human participants or groups of humans to one or more health-related interventions to evaluate the effects on health outcomes. Interventions include but are not restricted to drugs, cells and other biological products, surgical procedures, radiological procedures, devices, behavioural treatments, process-of-care changes, preventive care, etc ([3](#_ENREF_3)).

*Data custodian*

Research group, company, organisation, or sponsor that collects, manages and stores data from a clinical trial and would be responsible for data sharing.

*Data requester*

Researcher, or research group, that requests access to data from a clinical trial for the purpose of undertaking scientific research.

*Data sharing*

Defined by the Institute of Medicine as “the responsible entity (“data generator”) making the data available via open or restricted access, or exchanged among parties.”([4](#_ENREF_4))

N.B. Throughout this document the term ‘data custodian’ is used instead of ‘data generator’ to describe the entity responsible for sharing the data.

*Individual Participant Data (IPD)*

Individual Participant Data (IPD) are the data recorded within a clinical trial dataset associated with individual participants. This may be in the form of measurements of patient characteristics (weight, blood pressure, heart rate etc.), a description of a patient’s medical history and data collected about an individual participant’s clinical outcome during the trial. It can also include clinical laboratory results or images such as X-rays, details of randomisation and treatment received, and any adverse event information.

# Summary of Good Practice Principles for Sharing Individual Participant Data (IPD) from Publicly Funded Clinical Trials

The following is an overall summary of the good practice principles which are described in further detail in section 4.

| **Good practice at the CTU Level**  *Policy*   - - A data sharing policy should be developed by the CTU outlining the general approach to data sharing, summarising the elements discussed below. The CTU policy should align with any other overarching policies e.g. CTU’s host organisation policy, funder policies   *Scope*   - - IPD and associated documentation should be made available for all prospective publicly funded clinical trials. Requests for data from historical clinical trials should be dealt with on a case by case basis   - IPD should be made available as soon as reasonably possible e.g. 18 months after trial completion   *Data request process*   - - Sponsor approval for data sharing should be sought (sponsor might initially agree principles of data sharing but delegate responsibility for implementing data sharing to the CTU)   - Only bona fide research groups should be eligible to access data (e.g. evidenced via CVs and the involvement of a qualified statistician)   - Data access requests should be made via an application form detailing the specific requirements and the proposed research and publication plan   - Data access requests should be reviewed against specific eligibility criteria by data custodians (e.g., trial statistician and Chief Investigator) or by an external Independent Review Panel. Decisions about requests should be made promptly according to a published schedule (no more than 3 months after receipt of request)   - Details of all data requests and their outcomes, with clear rationale for any refusals, should be made publicly available (e.g. on the CTU website). Data requesters should be informed of this in advance   *Data release process*   - - Data should be made available as soon as possible after approval of requests   - Data should be made available on a secure server or via other secure data transfer method   - Supporting documentation should be supplied with the dataset   *Data use agreement*   - - A data use agreement should be utilised which, at a minimum,     1. Prohibits attempts to re-identify or contact trial participants     2. Addresses any requirements regarding planned outputs of proposed research e.g., publication and acknowledgement requirements     3. Prohibits non approved uses or further distribution of the data   *Resources*   - Funds for responsible data sharing should be requested from trial funders as part of initial trial grant applications e.g. to fund dataset preparation and anonymisation - Reasonable costs may be recovered from data requesters if appropriate but data sharing activities should not be profit generating - Host organisations (e.g., Institute of Higher Education) may be able to provide funds for routine data sharing activities e.g., ongoing maintenance of a data sharing system - Responsibilities of staff for data sharing should be determined and funding should be sourced |
| --- |
| **Good practice at the individual trial level**  *Prior to trial funding*   - - Identify data sharing stakeholders for a trial early on (e.g. Sponsor, Funder, Chief Investigator, Trial Management Group, CTU) and highlight the CTU data sharing policy   - Understand the trial funder’s policy and include plans and reasonable costs (if appropriate) for sharing IPD within the trial grant application   *During trial set-up*   - - Identify roles and responsibilities for data sharing activities and include on a delegation log   - Include outline plans for data sharing in the protocol (see SPIRIT checklist item 31c ([5](#_ENREF_5)))   - Include detailed plans for data sharing in the trial data management plan   - Include a data sharing statement in the consent form and information in the patient information leaflet. The Health Research Authority ([6](#_ENREF_6)) currently recommend the following wording:   “I understand that the information collected about me will be used to support other research in the future, and may be shared anonymously with other researchers.”   - - Annotate the complete set of blank Case Report Forms (CRFs) so that they clearly describe the data variable labels and values contained within the electronic dataset. This may not be required if dataset specifications and blank CRFs are sufficiently detailed to enable matching of data variables from CRFs to the electronic dataset (Note: blank CRFs made available on the CTU website, or some other forum, would help researchers identify relevant data that have been collected prior to submitting a formal request for data)   *End of trial*   - - Prepare the anonymised dataset ready for sharing. The level of anonymisation should be determined in conjunction with other considerations such as original patient consent and method of data transfer   - Dataset preparation should be done by individuals with an understanding of data management and basic statistics, with quality control provided by a further individual who is independent of the process   - Prepare ‘data pack’ ready for sharing. This would typically include (i) electronic datasets in a suitable format that is recognised by a range of statistical software, that could be easily divided to create a subset of data if required for the use case requested, and (ii) supporting documentation (minimum requirement would be protocol with amendments, blank CRFs, dataset specifications including data variable amendments). Timing of data pack preparation may be reactive or proactive. |

# Background

There is no dispute that the data generated from clinical trials can be used to address many important research questions beyond those planned in the original trial. This has the potential to provide real benefit to patients and the scientific community. Despite this, the large quantities of data generated throughout the conduct of a clinical trial have historically been considered “private property”; kept by either the trial sponsor or the clinical trial research group. As a result of this practice, further use of the data has been restricted to that original research group or, worse still, not used at all.

With the issue of sharing data from clinical trials in the spotlight, there has been considerable focus on how industry, regulatory bodies, clinical trial funders and sponsors can amend their practices to facilitate clinical trial data sharing. Barriers to data sharing are often presented without discussion of how to overcome them, and although several stakeholders are actively encouraging data sharing activities there are still more opportunities to increase the provision of data to other researchers for further use.

In our recent survey ([1](#_ENREF_1)), publicly funded CTUs from the UKCRC registered network of CTUs were supportive of the principle of sharing IPD but, because of the complex and varied sponsorship arrangements of the trials they coordinate, some CTUs did not think that data sharing should be their responsibility. Furthermore, the CTUs were commonly concerned about the inappropriate reuse of clinical trial data, the additional resource required for CTUs to prepare and share data, the potential loss of ability to publish further research, and the potential risk to trial participant privacy. None of the CTUs supported the use of an open access model to share IPD. Instead, a controlled access approach, with systems in place to review data access requests from researchers, was preferred. We have used the results of this survey, input from an expert committee (Appendix 4) and an open consultation involving the UKCRC registered CTUs to inform the development of this guidance.

## Aim

This document aims to provide guidance and good practice principles for sharing IPD and associated documentation from publicly-funded clinical trials conducted in the UK.

## Scope

The guidance document is aimed primarily at UK publicly funded Clinical Trials Units (CTUs) responsible for coordinating and storing data collected in clinical trials, but several principles will be applicable across the wider clinical trial community. An assumption is made throughout that all clinical trials should be registered and summary-level trial results published and made available in a timely manner; this guidance does not specifically address these issues. Instead the guidance focuses on the practicalities of making the underlying individual participant data (IPD) from a clinical trial available for scientific research purposes. The document does not cover issues specific to sharing human samples.

## Funder requirements

The two largest non-commercial funding bodies of UK based clinical trials are the MRC and the NIHR. The NIHR does not have a specific data sharing policy but does have a statement on open access that makes reference to research data. The Organisation for Economic Co-operation and Development (OECD) ([7](#_ENREF_7)) and Research Councils UK (RCUK) ([8](#_ENREF_8)) describe data sharing principles that should apply to all publicly funded research. The latter stipulates that from April 2013 any trial funded by a UK research council is obliged to include a statement in any published paper clarifying how and on what terms any supporting material, including data, may be accessed (or justification why data cannot be made available). These principles are reflected in the MRC Policy and Guidance on Sharing of Research Data from Population and Patient Studies ([9](#_ENREF_9)) which states:

“The MRC expects valuable data arising from MRC-funded research to be made available to the scientific community with as few restrictions as possible so as to maximize the value of the data for research and for eventual patient and public benefit. Such data must be shared in a timely and responsible manner.”

Other funders of clinical trials often align with the OECD and RCUK principles and funders often require applicants to submit plans for data management and sharing as part of the grant application. Some funding bodies will provide funds specifically for sharing information at the end of the trial.

## Legal requirements

“In the UK, the confidentiality of personal information is addressed primarily in Common Law. The Data Protection Act (DPA) 1998 ([10](#_ENREF_10)) superimposes on this a framework of rights and duties and principles governing the use of information in electronic form or structured paper records.” ([9](#_ENREF_9))

The DPA defines personal data as “data which relate to a living individual who can be identified: from those data; or from those data and other information which is in, or is likely to come into, the possession of the data controller”([10](#_ENREF_10)). With consented research data, the data controller’s main duty is to ensure that the data is used as consented; data subjects’ confidentiality is respected; and that data remains secure wherever it is held.

As a special case, by anonymising data to a form which does not identify individuals and where identification is not likely to take place, it is possible to provide greater access to individual level clinical trial data in a manner that protects individual privacy and confidentiality. While the legal requirements of the DPA do not apply to anonymised data it is still important to consider ethical issues such as whether requests to re-use anonymised data are broadly aligned with the purposes for which the data were collected. Approaches have been developed to anonymise clinical trial data for sharing whilst still protecting the privacy and confidentiality of participants ([11](#_ENREF_11)). Further details are provided in section 4.2.3.

## Sponsor requirements

An issue for publicly funded CTUs is the perception that the data do not ‘belong’ to them -they are the custodians of the data and are generally delegated the tasks of data collection, maintenance and archiving, but the sponsor is ultimately responsible for the trial and the data that has been generated. Therefore, the clinical trial sponsor should be involved in the process of sharing IPD, but the practicalities of actually doing this will most likely fall to the CTUs, who are in an excellent position to facilitate and promote better sharing.

## Regulatory requirements

Regulatory agencies have been making changes to promote transparency in clinical trials. The European Medicines Agency (EMA) adopted a new policy regarding publication of clinical study reports in October 2014 that mandates publication of clinical study reports supporting applications for centralised marketing authorisations ([12](#_ENREF_12)). The policy entered into force on 1^st^ January 2015.The EMA plans to also make IPD available from these trials at some point in the future following a consultation process but as yet there is no regulation mandating the sharing of IPD. In the US, the Food and Drug Administration (FDA) have also proposed making pharmaceutical product applications more transparent. In collaboration with the National Institutes of Health the FDA launched the Transparency Initiative in 2009 ([13](#_ENREF_13)) which aims to make de-identified and ‘masked’ patient-level data from clinical trials available to the public.

# Data Sharing Models

There are several models for providing access to clinical trial data which can be broadly separated into two categories of ‘open access’ and ‘controlled access’.

## Open access

In an open access model the anonymised IPD is made available to researchers and the public alike; no approvals are required and there are no limitations or restrictions on the use of the data. There are examples of research groups that have made IPD from clinical trials available in an open access system:

- IST-1 ([14](#_ENREF_14))

Anonymised IPD were published as supporting material in Trials, an open access journal. The research group addressed issues of acknowledgement, consent and the anonymisation process within the body of the article, and felt the sharing of this anonymised data presented minimal adverse risk to trial participants.

- FreeBIRD ([15](#_ENREF_15))

Anonymised datasets and data dictionaries from the CRASH-2 trial are openly available on the FreeBIRD (Free Bank of Injury and emergency Research Data) website of the London School of Hygiene and Tropical Medicine. Those wanting to access the data must register and validate an email address to obtain a username and password, but information about the purpose of the research is not required.

These examples demonstrate that the publication of anonymised IPD using an open access model is potentially possible. However, individual participants within a trial are at greater risk of being re-identified if data are released using an open access model as there are no restrictions in place and data may be abused or inappropriately merged with other data to facilitate re-identification. In terms of research governance, making data available via open access also makes it very difficult to track the publications that may arise from the original data and to monitor whether research using the data is being fully reported. This approach to data sharing may therefore inadvertently encourage selective reporting of secondary research using the data. It would be difficult, if not impossible, for researchers to discover whether similar work using the dataset is being undertaken at the same time which may reduce efficiency.

Therefore, whilst the open access system may appear to be the least burdensome and bureaucratic, this guidance recommends the use of a controlled access approach for sharing data with external researchers to facilitate research that may benefit scientific understanding and patient care. None of the UK CRC registered CTUs in our survey supported the use of an open access model ([1](#_ENREF_1)).

## Controlled access

In the controlled access model (Figure 1) data requesters have to provide information to support a request for data access. This information is reviewed against pre-specified criteria before a decision is made. This review can be undertaken internally (e.g. by a committee of members of the CTU and sponsor) or by an independent review committee for a fully unbiased approach as recommended by the Institute of Medicine ([16](#_ENREF_16)). Access to data may be granted but further restrictions regarding the access process may apply e.g. access through a secure web interface.

*Data Requester (e.g. researcher wishing to access IPD for research project) Tasks*

*Data Custodian (e.g. CTU) Tasks*

Complete access request

Provide supporting documentation e.g. research proposal

Review request against pre-specified criteria. This may be done by an independent review committee

If approved, prepare Data Use Agreement

Make data available

Sign Data Use Agreement

**Figure 1. Flow chart of activities in a controlled access model**

Two examples of a controlled access model using internal and external review processes for sharing IPD are provided in Case Study 1 and 2 below. Other possibilities exist, and have been explored extensively by e.g. the Economic and Social Research Council (ESRC) ([17](#_ENREF_17)) and the Wellcome Trust ([18](#_ENREF_18)) in distributing sensitive social science and genomic data, which both place more emphasis on data sharing through community-endorsed and funder-sponsored repositories.

| **Case Study 1: Internal review process (in house review of requests)**  **Keele CTU - Data Custodian and Academic Proposals (DCAP) Committee**  The Research Institute for Primary Care and Health Sciences at Keele University has an internationally renowned research programme utilising observational long-term cohorts with linked medical records, randomised clinical trials and linked qualitative research data sets. External researchers are encouraged to develop and lead research proposals for secondary analysis of the anonymised IPD collected in these studies, with the understanding that the analysis will be conducted in collaboration with Institute researchers. The Keele CTU, based within the Institute, have developed a process of internal review to approve and then manage the data release processes associated with requests to share data ([19](#_ENREF_19)).  External researchers are first invited to contact the Keele CTU to discuss their research proposal. The CTU, in conjunction with the Keele study PI, gives an initial assessment of scientific feasibility, and ensures the proposal does not duplicate ongoing or planned analysis within the study concerned. At least one of the original Keele study team members will be named as a collaborator on the planned project. The external researcher then completes an External Data Request Form. This includes detail of the research questions and the analysis proposed, as well as CVs of the external researchers, the plans for secure storage of the data, and details of the study population and variables required. The external researcher is also required to sign a data sharing agreement including that the data will be used only for the purposes of the proposal outlined in the request form and within the principles of the Data Protection Act, the Research Governance Framework, ethics approvals and the consent arrangements agreed for the use of the data. The data request is peer reviewed by the Keele Data Custodian and Academic Proposals (DCAP) Committee (consisting of senior CTU staff members including the CTU Co-Directors), alongside the PI and Lead Statistician of the original research team (if they are not members of the DCAP), to assesses feasibility and scientific quality of the proposal. If the request is approved, the form is signed by the Keele PI of the study from which data is requested and the study Data Custodian prepares the required datasets for provision to the external requester via a suitable method e.g., NHS.net or a password-protected Dropbox facility. If a request if not approved the DCAP Chair will contact the external researcher to explain the decision and discuss possible revisions.  Keele does not charge for or receive any specific funding to support its data sharing activities. At present research data sharing proposals are prompted by the initial study publication (see <http://www.keele.ac.uk/pchs/publications/datasharingresources/>). Keele hopes to identify funding sources to support a dedicated data manager to promote, expand and support its data sharing arrangements. |
| --- |

| **Case Study 2: External review process (independent review of requests)**  [**www.clinicalstudydatarequest.com**](http://www.clinicalstudydatarequest.com) **(**[**20**](#_ENREF_20)**)**  This website is currently utilised by GSK and other pharmaceutical companies to provide researchers with the opportunity to request anonymised clinical trial data to conduct further research. The site currently lists over 1300 clinical trials to which researchers can request access. Many of the sponsors also provide an enquiry option allowing researchers to ask for access to unlisted studies. To join the system and list trials, individual sponsors are required to provide information about their policy for listing studies, e.g., which studies are listed and reasons for any exclusions, the anonymisation standard for provided data, additional conditions for access and what datasets and documentation can be made available e.g., raw datasets, annotated Case Report Forms, protocols, Clinical Study Reports etc. Sponsors are able to define their own commitment (e.g. future and/or older studies) while using a common pathway for submitting requests, reviewing research proposals and accessing data.  Researchers must submit research proposals that are reviewed by an Independent Review Panel (IRP). Requests are reviewed against several criteria such as the scientific rationale of the proposed research, the publication plan and the qualifications and experience of the research team to conduct the proposed research (a research team should be supported by a statistician). When proposals are approved, a Data Sharing Agreement must be signed by the requester and relevant sponsors before access is granted. Data must be analysed within a secure access environment, which includes statistical software (SAS and R). Researchers are provided with access to support facilities (e.g. online help and user guides) to help them navigate the secure environment and the available datasets.  Both the request site and the access environment are provided by third party vendors and as such there are annual financial costs (paid by the sponsors) involved in both listing studies and providing access to data. The vendors offer tiered pricing structures to academic sponsors which vary based on the number of studies listed and the use of the access environment (i.e. the number of approved research projects and datasets made available). Efforts to continue to develop solutions that meet the needs of non-industry sponsors are ongoing. At present an academic sponsor would need to pay in the region of £10-20k annually to list 20 studies and provide access to 10 research projects. |
| --- |

# Good Practice Principles for Sharing Individual Participant Data (IPD) from Publicly Funded Clinical Trials

There is a strong case that there is an obligation to make the data arising from publicly-funded clinical trials available to other researchers to maximise the potential of the data. As such, this document supports the practice of making data available in a responsible manner whilst protecting the privacy and confidentiality of trial participants.

There are key data sharing activities that a CTU may have to consider throughout the clinical trial process (Figure 2) to ensure responsible data sharing. Activities have been separated into CTU level (which apply across multiple trials within the CTU portfolio) and trial level activities (which are specific to an individual trial although likely to be similar across different trials). An assumption is made throughout that the CTU is a data custodian and the sponsor has ultimate responsibility for authorising the release of data. A controlled access approach is assumed.

CTU Data Sharing Policy

Long Term Data Storage

Pre-trial

Trial set-up

End of trial

Anonymisation

CRF Annotation / database specifications

Consent Form

Funding

Set up Access and Review Process

Stakeholders

Data Management Plan

Data Use Agreement

Data pack preparation

Protocol

Data Provision

Key:

Consideration at the CTU level

Consideration at the individual trial level

**Figure 2. Data sharing activities through a clinical trial process**

## Considerations at the CTU level - Data Sharing Policy

The purpose of a data sharing policy is to define policy and procedures within a CTU to ensure that research data can be effectively re-used by others ethically, legally and within commercial constraints. Development of a policy allows CTUs to justify their position on data sharing as well as describing the datasets that may be available and the mechanisms of access. Having a data sharing policy will increase transparency and improve clarity for future researchers wishing to access data. A CTU data sharing policy should also consider other relevant overarching policies including requirements of journals where trial results would be published, requirements of trial funders and the sponsor organisation’s policy on data sharing.

Develop a CTU data sharing policy

When developing a data sharing policy the following should be considered:


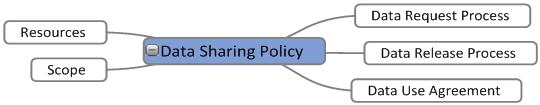


### Scope

The data sharing policy should outline the scope of trials for which IPD will be made available. This should specify that IPD and associated documentation will be made available for all future publicly funded clinical trials and requests for data from historical clinical trials dealt with on a case by case basis. It may be unreasonable to expect CTUs to include clinical trials for which only a peripheral role has been taken (i.e. if the CTU is not taking the lead for coordinating and storing data from a clinical trial). Details should also be provided about the expected timescale for making trial data available. The Institute of Medicine has recently published a recommendation that “It is reasonable to expect clinical trial data that will not be part of a regulatory application to be available for sharing no later than 18 months after study completion. This may not be realistic in some trials where trial participants are followed up beyond the time of primary analysis”([16](#_ENREF_16)). A period of 18 months should provide sufficient time for the trial team to complete and publish all planned primary analyses. Nevertheless, providing a reasonable definition of when IPD would be available is good practice.

Define the scope of data for sharing

### Data request process

A data sharing policy should include details of the data request process to ensure transparency and clarity. As publicly funded CTUs are not generally the sponsor for the trial, it is necessary to define a process for obtaining sponsor approval for data sharing. The most efficient process will be to seek overall trial level approval from the sponsor with implementation of processing individual data requests and data release delegated to the CTU.

Define the data request process

Further suggested details that define the data request process include:

- - Who is eligible to request IPD (e.g. only qualified research groups).
  - How requests for IPD are made (e.g. through CTU website; via a specific e-mail address).
  - Information required from the data requester to facilitate review (e.g., research proposal including statistical analysis plan, publication plan and evidence of research group qualifications). An example is provided in Appendix 1.
  - What criteria will be used for making decisions about access to IPD (e.g., scientific considerations of the proposed research, adequate qualifications of research group).

Define the criteria and process for authorising access

- - Who will be involved in the review process (e.g., Trial Management Group and Chief Investigator or an Independent Review Committee as recommended by the Institute of Medicine ([16](#_ENREF_16))) and how the process would work for historical trials where the original team have disbanded.
  - Estimated length of time to reach a decision about a data request (e.g., within 3 months of receiving request) recognising that additional time may be required to prepare and release the data.
  - How data requests and their outcome will be recorded, communicated to data requesters, and whether appeals are permitted – a fully transparent system would make information available about all data requests and their outcomes, including reasons for refusals, publicly available.

Define the process of releasing data

### Data release process

Following approval of a data request, the release of data should occur as soon as possible. Supporting documentation should be supplied with the dataset (see 4.2.3). Data release via a secure transfer method can occur in a number of ways as determined most appropriate by the data custodian. Possible approaches include direct transfer of encrypted anonymised datasets to the data recipient or dataset upload to a restricted access repository. Several repositories already exist and CTUs may consider using these for sharing data. For example, FigSHARE ([21](#_ENREF_21)) is a cloud-based repository supported by Digital Science that can host any file type from protocols to full datasets. There is an option to upload data into private or collaborate spaces which allows controlled access via private links that can be removed at any time. Data are easily searchable, citable and trackable.

### Data use agreement

Employ a data use agreement

It is essential that data sharing is based on trust and mutual understanding from all parties. Data use agreements, which can come by many terms such as “data sharing agreement”, “access agreements” and “data transfer agreements”, are legal documents that detail the various conditions and responsibilities that must be agreed to by both parties and should include details of:

- - The data custodian’s expectations of the data requester (e.g., to prohibit attempts to re-identify participants in anonymised datasets, required action if re-identification occurs, prohibits sharing of the data outside of the research team, agreement to conduct and publish research as outlined in original research plans).
  - Requirements for ensuring datasets are returned, destroyed or access terminated at the end of a research project.
  - Consequences of non-compliance (e.g., revoking access or legal action).
  - If appropriate, how the original trial team and funder should be acknowledged in projects and future publications that use the trial data (e.g. co-authorship of trial team, acknowledgement for use of trial data or reference to Digital Object Identifier (DOI) for the data).

Data use agreements provide the data custodian with additional assurances of patient privacy protection and responsible data use but also highlight essential responsibilities to the data requester. An example of a simple data use agreement template is provided in Appendix 1 and an example of a more detailed template has been developed by the Multi-regional **Clinical Trials Center at Harvard University (**[**22**](#_ENREF_22)**).**

### Resources

Consider resources and responsibilities for data sharing

A CTU should consider how data sharing activities will be funded and who will be responsible for undertaking the activities. The main additional resources required for data sharing will be for administering and reviewing data requests, preparing data and distributing data. All other activities (e.g. CRF annotation/creating dataset specifications) are considered to be good practice as part of any trial process and should not require additional resources. Reasonable data sharing costs (e.g. for data management and statistician time to create anonymised datasets) can be legitimately included in a trial grant application if funders are willing to support this activity. Alternatively, CTUs could recover limited reasonable costs from data requesters if absolutely necessary, noting that data sharing activities should not be profit generating. The resources required are likely to vary for each trial and a combination of approaches may be appropriate. Consideration should be given to the resources required for the ongoing maintenance of a data sharing system at the CTU - this may be most appropriate for the organisation hosting the CTU to provide.

## Considerations at the individual trial level

Identify data sharing stakeholders as early as possible

At the individual trial level the following good practices can facilitate the data sharing process and increase transparency.

### Prior to trial funding

Data sharing activities should be considered early on in the trial process, ideally prior to submitting a trial funding application. The CTU policy for sharing data should be highlighted to stakeholders involved in a trial e.g. Chief investigator, funder, sponsor(s), Trial Management Group etc. It is important to understand the trial funder’s requirements and, if appropriate, include plans and reasonable costs for future sharing of IPD within the grant application. Indeed, many funding bodies (e.g. MRC, Cancer Research UK) request specific details of data sharing plans to accompany research proposals.

Include costs for data sharing in trial grant application

### During trial set-up

Identify roles and responsibilities

The roles and responsibilities for data sharing should be identified and included in the delegation of duties. In addition the outline plans for data sharing should be included in the protocol as recommended by the SPIRIT checklist, item 31c ([5](#_ENREF_5)). Further detailed information can be included within the trial’s data management plan.

Include plans for data sharing in protocol and data management plan

An important consideration during trial set-up is the issue of participant consent for data sharing. The best way to alleviate potential ethical issues related to future data sharing is to obtain consent from participants to share their data for the purposes of scientific research. The Health Research Authority (HRA) ([6](#_ENREF_6)) currently suggests the following wording to be used in Participant information sheets and consent forms:

Request consent from trial participants to share their IPD

“I understand that the information collected about me will be used to support other research in the future, and may be shared anonymously with other researchers.”

There are many examples of anonymised IPD being shared with external researchers, and in the majority of cases specific consent for these activities will not have been obtained. Although good practice, a lack of consent for sharing should not prohibit sharing as anonymised data that it is not "reasonably likely" to lead to the identification of individuals when matched with data available elsewhere, will fall outside of the scope of the Data Protection Act ([10](#_ENREF_10)). Unfortunately, it can be difficult to predict the risk of re-identification through data linkage, and the security measures provided by a controlled access system can provide additional assurance of participant privacy protection.

Annotate the complete set of blank Case report Forms or create dataset specifications

A final consideration during trial set-up is to annotate the complete set of blank Case Report Forms (CRFs), or create dataset specifications which clearly map the data fields from the blank CRF to the electronic database. CTUs may further consider publishing their blank CRFs (e.g. on the CTU website) to help researchers identify relevant data that have been collected to further aid research and increase transparency.

The use of common standards and processes for annotating data across clinical trials could further simplify future data sharing, although this is not a necessity. Standardisation during the initial data collection could make the upload of clinical trial data onto multi-sponsor repositories much easier, and also facilitate the combination of datasets for meta-analysis without considerable extra manipulation. Researchers will be instantly familiar with other datasets and will be able to navigate through the datasets efficiently, reducing errors arising from misinterpretation. In the US, it is mandated that clinical trial data submitted to the FDA for regulatory purposes are collected and maintained using standards described by the Clinical Data Interchange Standards Consortium (CDISC) ([23](#_ENREF_23), [24](#_ENREF_24)). Currently, there is no such mandate in the UK but it is possible that the EMA will take the advice proposed by their clinical trial advisory groups and make attempts to harmonise clinical trial data standards with those of the FDA ([25](#_ENREF_25)) in future. However, CDISC can be difficult to apply and adapt and requires investment in specialised data management resources. In our survey of CTUs ([1](#_ENREF_1)) only 10% of responding CTUs said that CDISC was currently being used.

### End of trial

At the end of a trial, e.g. when all participant data have been collected and the database frozen, the main actions for the data custodian will be to prepare the data ready for sharing. It may be most efficient to undertake this activity routinely at the end of each trial when the individuals who are most familiar with the trial and the data are still available. Alternatively, this could also be done in response to a direct request for data. The following should be considered:

- **Prepare the anonymised dataset prior to sharing**

Prepare anonymised dataset for sharing

Protecting participant privacy and confidentiality is the over-riding consideration when sharing IPD. In some cases this can be achieved by anonymisation, a process of turning data into a form which does not identify individuals and where identification is not likely to take place ([2](#_ENREF_2)). Anonymisation can also remove legal constraints of the Data Protection Act ([10](#_ENREF_10)).

There are many different techniques for anonymising data that may be used individually or in combination to minimise the risk of patient re-identification within a dataset. To be considered fully ‘anonymised’ there are certain identifying characteristics that must be removed or replaced. Hrynaszkiewicz *et al* ([11](#_ENREF_11)) have formulated a list of 28 patient identifiers based on information aggregated from policy documents and research guidance from major UK and US funding agencies, governmental health departments and statutes, and three internationally recognised publication ethics resources (Appendix 2). This list includes the 18 identifiers detailed in the Privacy Rule applying to “Protected Health Information” of the US Health Insurance Portability and Accountability Act (HIPAA) ([26](#_ENREF_26)). The anonymisation approaches adopted by study sponsors using www.clinicalstudydatarequest.com have been published on the website – they are broadly similar to the GSK approach described in ([27](#_ENREF_27)) and Appendix 3. Further examples of good practice are provided by the UK Data Archive ([17](#_ENREF_17)) and the Information Commissioner’s Office ([2](#_ENREF_2)).

It is important to note that some anonymisation techniques could limit the scope and utility of possible secondary analyses. For example, removing text variables that describe Adverse Events and Medications or removing dates of events may weaken datasets substantially and these approaches may be most suitable for completed trials where specific consent for sharing data has not been obtained. Looking to the future, if trial participants have provided consent for their data to be shared for research purposes (recommended in section 4.2.2), and if data are being shared within the confines of a controlled access system with data use agreements and strict governance procedures, then less stringent approaches to anonymising trial datasets may be appropriate in order to maintain the richness of data. The Institute of Medicine discuss assessing the trade-off between privacy and data utility (([16](#_ENREF_16)), p197-8) which would allow the degree of anonymisation to vary. As an example of how this works in practice, the ESRC funded UK Data Archive ([17](#_ENREF_17)) has implemented a generic, three tier access policy that combines modes of access and conditions of use which are determined according to the residual risk of disclosure and the level of sensitivity of particular social and economic data. Suitable infrastructure to secure systems which allow data access but restrict data download would likely be required to support such a model, and this may be something for the trial community to work towards in the future.

Final anonymised dataset should be reviewed by a third party

Data should be anonymised by data custodian team members who have an understanding of data management and basic statistics and preferably with some knowledge of the trial. Once data have been anonymised, appropriate internal quality control and assurance steps should be undertaken to thoroughly check the final anonymised data before it is released. If attempting full anonymity, it is good practice to use re-identification testing, as described further by the UK Information Commissioner’s Office ([2](#_ENREF_2)). Otherwise, data should be checked against the Hrynaszkiewicz et al ([11](#_ENREF_11)) checklist (Appendix 2) to confirm that data items that are not anonymous individually or in combination, have not been left in the dataset inadvertently.

- **Prepare ‘data pack’ ready for sharing**

Prepare ‘data pack’ ready for sharing

It is important to provide adequate supportive information to allow researchers to interpret the data accurately. This will minimise the risk of incorrect secondary analysis and reduce the number of queries received by requesters trying to use the data. The suggested content of the data pack is listed in Table 1.

**Table 1. Suggested content of the data pack ready for sharing**

| **Suggested content of data pack** | **Description** |
| --- | --- |
| Anonymised data * | Electronic data collected for each patient in the trial in a format that can be recognised by a wide range of statistical software (e.g. SAS, Stata, R). The use of ‘StatTransfer’ or other similar product may be useful for this purpose. |
| Blank CRF * | Blank CRFs with descriptions of the data collected. These could be annotated to provide a map of the data variables within the dataset, or provided as blank CRFs along with dataset specifications |
| Dataset specifications* | Meta-data describing the datasets e.g., data freeze date, variable labels, variable descriptions, formats, and summary of amendments made during the trial e.g., changing data definitions, adding/removing variables |
| Protocol * | Trial protocol, including all amendments |
| Statistical Analysis plan | Methods of analysis and procedures for data handling used in the final statistical analysis (this is useful if researchers want to replicate published analyses to facilitate their understanding of the dataset) |
| Analysis programs | Programs used for generating and analysing data used in the final analysis report (this is useful if researchers want to replicate published analyses to facilitate their understanding of the dataset) |
| Clinical Study Report (CSR) (or equivalent) if applicable | Report of efficacy and safety data from the trial that forms the basis of submissions to regulatory authorities e.g., EMA |
| * Essential content |  |

# References

1. Hopkins C, Sydes M, Murray G, Woolfall K, Clarke M, Williamson P, Tudur Smith C. Clinical trial data sharing and access from publicly-funded Clinical Trials Units’ perspectives: an opinion survey. 2015;Submitted

2. Information Commissioner's Office. Anonymisation: managing data protection risk code of practice. 2012.

<https://ico.org.uk/media/for-rganisations/documents/1061/anonymisation-code.pdf>

3. World Health Organisation. <http://www.who.int/topics/clinical_trials/en/>

4. Institute of Medicine. Discussion Framework for Clinical Trial Data Sharing: Guiding Principles, Elements and Activities. 2014. [http://www.iom.edu/Reports/2014/Discussion-Framework-for-Clinical-Trial-Data-Sharing.aspx](http://www.iom.edu/Reports/2014/Discussion-Framework-for-Clinical-Trial-Data-Sharing.aspx:)

5. Chan A-W, Tetzlaff JM, Altman DG, Laupacis A, Gøtzsche PC, Krleza-Jeric K, et al. SPIRIT 2013 Statement: Defining Standard Protocol Items for Clinical Trials. Annals of Internal Medicine. 2013;158(3):200-7.

6. Health Research Authority. <http://www.hra.nhs.uk/resources/before-you-apply/consent-and-participation/consent-and-participant-information/>

7. Organisation for Economic Co-operation and Development. OECD Principles and Guidelines for Access to Research Data from Public Funding. 2007. <http://www.oecd.org/science/sci-tech/38500813.pdf>

8. Research Council UK. RCUK Common Principles on Data Policy. <http://www.rcuk.ac.uk/research/datapolicy/>

9. Medical Research Council. MRC Policy and Guidance on Sharing of Research Data from Population and Patient Studies. 2011. <http://www.mrc.ac.uk/news-events/publications/mrc-policy-and-guidance-on-sharing-of-research-data-from-population-and-patient-studies/>

10. UK Parliament. Data Protection Act. 1998. <https://www.gov.uk/data-protection/the-data-protection-act>

11. Hrynaszkiewicz I, Norton ML, Vickers AJ, Altman DG. Preparing raw clinical data for publication: guidance for journal editors, authors, and peer reviewers. BMJ. 2010;340:c181.

12. European Medicines Agency. European Medicines Agency policy on publication of clinical data for medicinal products for human use. EMA/240810/2013. October 2014. <http://www.ema.europa.eu/docs/en_GB/document_library/Other/2014/10/WC500174796.pdf>

13. U.S. Food and Drug Administration. FDA Transparency Initiative. 2009. <http://www.fda.gov/AboutFDA/Transparency/TransparencyInitiative/default.htm>

14. Sandercock P, Niewada M, Czlonkowska A, International Stroke Trial Collaborative Group. The International Stroke Trial database. Trials. 2011;12:101.

15. London School of Hygiene and Tropical Medicine. FreeBIRD Bank of Injury and Emergency Data. <https://ctu-app.lshtm.ac.uk/freebird/>

16. Institute of Medicine. Sharing Clinical Trial Data: Maximizing Benefits, Minimizing Risk. 2015. <http://iom.edu/Reports/2015/Sharing-Clinical-Trial-Data.aspx>

17. UK Data Archive. <http://www.data-archive.ac.uk/>

18. European Genome-phenome Archive. <https://www.ebi.ac.uk/ega/>

19. Keele Clinical Trial Unit. <http://www.keele.ac.uk/pchs/keelectu/>

20. Clinical Study Data Request. [www.clinicalstudydatarequest.com](http://www.clinicalstudydatarequest.com)

21. FigSHARE. <http://figshare.com/>

22. Multi-regional Clinical Trials Center at Harvard University. 2015-03-25 MRCT Center Data Use Agreement Template. 2015. <http://mrct.globalhealth.harvard.edu/file/376776>

23. Food & Drug Administration. Study Data Standards for Regulatory Submissions Position Statement. 2013. <http://www.fda.gov/ForIndustry/DataStandards/StudyDataStandards/ucm368613.htm>

24. Clinical Data Interchange Standards Consortium (CDISC). <http://www.cdisc.org/>

25. Clinical Trial Advisory Group. Final advice to the European Medicines Agency from the clinical trial advisory group on Clinical trial data formats. 2013. <http://www.ema.europa.eu/docs/en_GB/document_library/Other/2013/04/WC500142850.pdf>

26. United States Department of Health & Human Services. Summary of the HIPPA privacy rule. 2003. <http://www.hhs.gov/ocr/privacy/hipaa/understanding/summary/privacysummary.pdf>

27. Hughes S, Wells K, McSorley P, Freeman A. Preparing individual patient data from clinical trials for sharing: the GlaxoSmithKline approach. Pharmaceutical Statistics. 2014;13(3):179-83.

# Appendix 1: Examples of Documentation

| **Details of trial being requested** | |  |
| --- | --- | --- |
| Title of trial and acronym: |  |  |
| Citation of trial publication (if relevant): |  |  |
| Trial registration number (if relevant): |  |  |
| **Data requester details (please attach full curriculum vitae of the research team)** | |  |
| Name of requester: |  |  |
| Position of requester: |  |  |
| Date of request: |  |  |
| Institute where research will be conducted: |  |  |
| Name/Title of scientific leader: |  |  |
| Institute of scientific leader: |  |  |
| Name/Title of Methodologist/Statistician: |  |  |
| Institute of Methodologist/Statistician: |  |  |
| Name/Title/position of any other research team members: |  |  |
| Institute of any other research team members: |  |  |
| **Description of the research project** |  |  |
| Title: |  |  |
| Background/rationale: |  |  |
| Objectives: |  |  |
| Endpoints, analysis plan and statistical  methods |  |  |
| **Practical details of the project** | |  |
| Expected start date: |  |  |
| Expected completion date: |  |  |
| Funding received or sought for the project: |  |  |
| **Data specifications** | |  |
| Which data may be required: |  |  |
| Other conditions: |  |  |
| **Project Outputs** |  |  |
| Expected number of planned publications: |  |  |
| Expected title(s) of planned publications: |  |  |
| **Signature of data requester:** |  |  |

**Example Data Request Form**

For an alternative example see: <http://www.ctu.mrc.ac.uk/13391/13399/data_sharing_application_form>

**Example Data Use Agreement**

**This agreement governs the terms on which access will be granted to the trial data detailed below. In signing this agreement the data requester is agreeing to be bound by the terms and conditions of access set out in this agreement. The terms of access set out in this agreement apply both to the data requester and the data requester’s Institution.**

Project title:

Identifier of the trial data being shared:

**In signing this Agreement:**

1. You agree to use the data only for the advancement of medical research, that access to the data is limited only to what is relevant to the completion of the project as described in the approved research proposal, and that all supplied data and any copies made thereof, will be destroyed at the end of the project.
2. You agree to use the data according to the terms specified in this Data Use Agreement. You accept that the data is protected by and subject to international laws, including but not limited to the UK Data Protection Act 1998, and that You are responsible for ensuring compliance with any such applicable law.
3. You agree to preserve the confidentiality of information and data pertaining to trial participants. You undertake not to use, or attempt to use the data to compromise or otherwise infringe the confidentiality of information on participants and their right to privacy.
4. You will not attempt to establish the identity of, or communicate with, any of the trial participants. You agree not to attempt to link the data provided under this agreement to other information, even if access to that data has been formally granted to you, without specific permission being sought from the [data custodian].
5. You agree not to transfer or disclose the data, in whole or part, to others outside the research team listed in the approved research proposal. You will require anyone listed in the research team who utilises these data to comply with the terms of this agreement.
6. You will refer to the [trial identifier] and acknowledge the [data custodian] in any publication arising from the use of these data using the following wording [*insert suitable wording to include acknowledgment of trial sponsor and funder*].
7. You will contact the [data custodian] if any safety concerns are identified during the project.
8. You will ensure use of appropriate administrative, physical and technical safeguards to prevent use or disclosure of the data other than as provided for by this Agreement.
9. You accept that if the conditions relating to the release of data as per the terms specified by the [data custodian] are knowingly disregarded that this will be considered a serious offence and could result in further action being taken the [data requester].
10. You understand that research using the data should be published according to the publication plan described in the approved research proposal.

**Data Requester Signature Data Requester’s Institutional Signature**

Signature: Signature:

Printed Name: Printed Name:

Position: Position:

Institution Name: Institution Name:

Date: Date:

# Appendix 2: List of 28 potential patient identifiers in datasets

*Reproduced with permission from Hrynaszkiewicz et al (*[*11*](#_ENREF_11)*)*

| Identifier | Comments |
| --- | --- |
| ***Direct*** | |
| Name |  |
| Initials |  |
| Address, including full or partial postal code |  |
| Telephone or fax numbers or contact information |  |
| Electronic mail addresses |  |
| Unique identifying numbers | Generalised HIPAA items 7-10, 18 |
| Vehicle identifiers |  |
| Medical device identifiers |  |
| Web or internet protocol addresses |  |
| Biometric data |  |
| Facial photograph or comparable image |  |
| Audiotapes |  |
| Names of relatives |  |
| Dates related to an individual (including date of birth) |  |
| ***Indirect—may present a risk if present in combination with others in the list*** | |
| Place of treatment or health professional responsible for care | Could be inferred from investigator affiliations |
| Sex |  |
| Rare disease or treatment |  |
| Sensitive data, such as illicit drug use or “risky behaviour” |  |
| Place of birth |  |
| Socioeconomic data, such as occupation or place of work, income, or education | MRC requirement is for “rare” occupations only |
| Household and family composition |  |
| Anthropometry measures |  |
| Multiple pregnancies |  |
| Ethnicity |  |
| Small denominators—population size of <100 |  |
| Very small numerators—event counts of <3 |  |
| Year of birth or age | Age is potentially identifying if the recruitment period is short and is fully described |
| Verbatim responses or transcripts |  |

# Appendix 3: Example Anonymisation Standard

This anonymisation standard has been provided by GlaxoSmithKline (GSK). Any updates to the GSK data anonymisation standard will be available at <https://clinicalstudydatarequest.com/Study-Sponsors-GSK-Details.aspx> (version accessed 01/2015)

**Anonymisation of Clinical Trial Datasets**

1. **Introduction**

Providing access to data in ways that allows further research while maintaining the privacy and confidentiality of research participants is critical. There are also privacy laws and regulatory guidance which need to be followed (for example [guidance from European data protection regulators](https://clinicalstudydata.gsk.com/Documents/Privacy-European-guidance.pdf) and [Code of Federal Regulations - Title 45: Public Welfare, Subtitle A §164.514](https://clinicalstudydata.gsk.com/Documents/Privacy-US-guidance.pdf)). Publications in this area which provide guidance^[[1]](#footnote-2)^,^[[2]](#footnote-3)^.

This document describes the approach taken by study sponsors to prepare data for sharing with other researchers in a way that:

- Minimises risks to the privacy and confidentiality of research participants.
- Ensures compliance with data privacy legal requirements.

Other study sponsors achieve these objectives using other approaches (see the Study sponsors section of ClinicalStudyDataRequest.com).

1. **General Approach**

Access is provided to anonymised data. Anonymisation involves:

1. **Removing personally identifiable information (PII) from the dataset.** This includes recoding identifiers (by replacing the original code number with a new code number), removing free text verbatim terms, Replacing date of birth with year of birth or age and replacing all dates relating to individual subjects with dummy dates or replacing them with a study day.
2. **Destroying the link (code key) between the dataset that is provided and the original dataset.** Some Data Protection Authorities in Europe suggest that the data can only be considered anonymised if personal information is removed (or redacted) and the subject code number cannot be linked to a research participant. Therefore, research participants’ identification code numbers are anonymised by destroying the code key that was used to generate the new code number from the original (i.e. destroying the link between the two code numbers).
3. **Removing personally identifiable information (PII) from the dataset**

The 18 identifiers (as defined by HIPAA –see [Code of Federal Regulations - Title 45: Public Welfare, Subtitle A §164.514](https://clinicalstudydata.gsk.com/Documents/Privacy-US-guidance.pdf)) are removed from the datasets (and related documentation). In addition any other PII that may be present is removed.

This involves removing:

- any names and initials,
- (or recoding) kit numbers and device numbers
- geographic information such as place of work.
- some sponsors also remove socioeconomic data such as occupation, income or education. Household and family composition. Multiple pregnancies.

In addition the following steps are undertaken:

- Recoding identifiers (or code numbers).
- Removing free text verbatim terms.
- Replacing date of birth with year of birth or age at randomisation**.**  Ages above 89 which are aggregated into a single category of “90 or older”. (This is a specific HIPAA requirement).
- Replacing all original dates relating to individual subjects with randomly generated offsets which are then applied to create ‘dummy dates’ or deleting these dates and replacing them with a study day. (see below)
- Reviewing and removing other PII

These steps are described in further detail below.

***3.1 Recoding Identifiers (or code numbers)***

The following identifiers (code numbers) are re-coded and the code key that was used to generate the new code number from the original code number is destroyed (as described in section 5):

- - The investigator identifier (or code number) is re-coded or set to blank for each investigator. The investigator name is set to blank or dropped from the dataset. (see Appendix 3a & 3b)
  - A new subject identifier (or code number) for each research participant.
  - Some sponsors also re-code the centre identification number.
  - Some sponsors aggregate patients from centres with less than 10 patients into a single centre.
- The same new identifiers (or code numbers) are used across all datasets applicable to a single study e.g. raw dataset, analysis-ready dataset. This includes (where applicable) PK datasets, genetic datasets etc.
- Extension studies use the same new identifiers (or code numbers) as used for the initial study to enable individual subject data to remain linked. This also applies to long term follow-up studies where separate reports are published. This is achieved by repeating the data anonymisation process for the initial study data at the same time as the extension/follow up data.
  1. ***Removing Free Text Verbatim Terms***

Information in a descriptive free text verbatim term may compromise a subject’s anonymity.

- Free text verbatim terms are set to “blank” or dropped from the dataset including:
- Adverse Events
- Medications
- Other e.g. Medical History
- Other specific verbatim free text

Certain free text fields may be retained if they do not contain PII and removal of these fields may impact the scientific value of the dataset (e.g. medical history that has not been coded).

- All dictionary coded terms with decode and/or verbatim terms that use a pre-specified list are retained.
  1. ***Replacing Date of Birth***

Information relating to a research participant’s date of birth and identification of specific ages above 89 may compromise anonymity.

- Date of birth is replaced with the year of birth or age at randomisation with the exception all of ages above 89 which are aggregated into a single category of “90 or older”

***3.4 Replacing all Original Dates relating to a Research Participant***

Study sponsors use one of two methods as described below.

- - 1. **Dummy Date Method**

Specific dates (other than year) directly related to a research participant may compromise a research participant’s anonymity.

All dates are replaced: A random offset is generated for each research participant and applied to all dates for that research participant. All original dates are replaced with the new dummy dates so that the relative times for each research participant are retained.

**Example:** If the original reference date was 01APR2008 and the date of death was 01MAY2008, a random offset is generated (in this case 91 days). Dummy dates are than calculated using this offset of 91 days.

|  | **Original Date** | **New Date** |  |
| --- | --- | --- | --- |
| Reference date | 01APR2008 | 01JUL2008 | Apply offset = 91 days |
| Date of Death | 01May2008 | 31Jul2008 | Apply offset=91 days |
| Relative Time of death | 30 days | 1. days |  |

- - 1. **Study Day Method**

All dates are removed from the datasets. The Study Day is calculated for each observation with days relative to a reference date. In order of priority the reference date is defined as the date of first study treatment, date of randomisation or date of consent. For example if a patient is randomised, but does not take the study treatment (i.e. the date of first treatment is missing), the date of randomisation will be used as the reference date to calculate the study day for any assessments recorded.

**Example** If the original reference date was 01JAN2008 and the date of death was 01MAY2008, the date of death would be 122 expressed as Study Days.

|  | **Original Date** | **Reference Date** | **Study Day** |
| --- | --- | --- | --- |
| Date of Death | 01May2008 | 01Jan2008 | 122 |

***3.5 Reviewing and Removing Other PII***

- Other data elements that contain PII are removed. For example:
- Information from variable names e.g. lab names may contain location information
- Investigator comments may be used to identify a subject
- Genetic data that would enable a direct trace back to an individual subject

Appendix 3a: Illustrates non-real examples of how these steps are applied.

1. **Review and Quality Control**

A final review of the HIPAA 18 identifiers is made to determine if further removal is required. Quality Control checks and documentation (QC record) is conducted for the processing of the data and supportive metadata documentation.

1. **Destroying the link (key code) between the dataset that is provided and the original dataset**

Research participants’ identification code numbers are anonymised by replacing the original code number with a new code number (as described in 3.1) and destroying the code key that was used to generate the new code number from the original (i.e. destroying the link between the two code numbers).

The following specific items are discarded:

- Any transactional copies of anonymised datasets
- De-identification tables (links for original variable and new anonymised variable)
- Any QC output datasets
- Any Log or LST files
- The seed utilised for random number generation

The anonymised datasets are stored in a separate secure location to the original coded datasets.

**Appendix 3a: A non-real example illustrating removal of personally identifiable information using the dummy date method**

| **Centre ID** | **Investigator ID (INVID)** | **Investigator name (INVNAME)** | **Subject ID (SUBID)** | **Unique subject ID (USUBID)** | **Age (yrs)** |  | **AE start date** | **AE end date** | **Verbatim term** |
| --- | --- | --- | --- | --- | --- | --- | --- | --- | --- |
| **00123** | **279344** | **Dr Smith** | **5** | **TJF4392.005** | **57** |  | **29DEC2010** | **27JAN2011** | **Headache** |
| **00123** | **279344** | **Dr Smith** | **2** | **TJF4392.002** | **72** |  | **10JAN2011** | **06APR2011** | **Nausea** |
| **00123** | **279344** | **Dr Smith** | **1** | **TJF4392.001** | **91** |  | **25MAR2011** | **12AUG2011** | **Cold** |
| **00123** | **279344** | **Dr Smith** | **66** | **TJF4392.066** | **89** |  | **28MAR2011** | **31MAR2011** | **Cold** |
| **00123** | **279344** | **Dr Smith** | **8** | **TJF4392.008** | **94** |  | **01MAR2011** | **15MAY2011** | **Flu** |
| **05678** | **333721** | **Dr Jones** | **19** | **TJF4392.019** | **85** |  | **14OCT2010** | **20OCT2011** | **Cold** |
| **05678** | **333721** | **Dr Jones** | **4** | **TJF4392.004** | **53** |  | **24MAY2011** | **.** | **Headache** |
| **05678** | **333721** | **Dr Jones** | **23** | **TJF4392.002** | **76** |  | **01MAR2011** | **15MAR2011** | **Pain** |

|  | **New INVID** | | **Remove INVNAME** | | **New SUBID** | | **New**  **USUBID** | | **Remove ages above 89** | | | **Create age category** | | **Add dummy dates** | | **Add dummy dates** | | **Remove** |  |
| --- | --- | --- | --- | --- | --- | --- | --- | --- | --- | --- | --- | --- | --- | --- | --- | --- | --- | --- | --- |
| **Centre ID** | | **Investigator ID (INVID)** | | **Investigator name** | | **Subject ID (SUBID)** | | **Unique subject ID (USUSID)** | | **Age (yrs)** | **Age Category** | | **AE start date** | | **AE end date** | | **Verbatim term** | | |
| **00123** | | **227** | |  | | **8754** | | **TJF4392.8754** | | **57** | **<=89** | | **19AUG2010** | | **17SEP2010** | |  | | |
| **00123** | | **227** | |  | | **5681** | | **TJF4392.5681** | | **72** | **<=89** | | **06JUL2010** | | **30SEP2010** | |  | | |
| **00123** | | **227** | |  | | **1475** | | **TJF4392.1475** | | **.** | **>89** | | **05SEP2010** | | **23JAN2011** | |  | | |
| **00123** | | **227** | |  | | **6589** | | **TJF4392.6589** | | **89** | **<=89** | | **06SEP2010** | | **09SEP2010** | |  | | |
| **00123** | | **227** | |  | | **3562** | | **TJF4392.3562** | | **.** | **>89** | | **29JUN2011** | | **12SEP2011** | |  | | |
| **05678** | | **208** | |  | | **1457** | | **TJF4392.1457** | | **85** | **<=89** | | **16JUL2011** | | **12SEP2011** | |  | | |
| **05678** | | **208** | |  | | **2214** | | **TJF4392.2214** | | **53** | **<=89** | | **04NOV2010** | | **.** | |  | | |
| **05678** | | **208** | |  | | **2236** | | **TJF4392.2236** | | **76** | **<=89** | | **01JUL2010** | | **15JUL2010** | |  | | |

**Appendix 3b: A non-real example illustrating removal of personally identifiable information using the study day method and aggregation of small centres**

| **Centre ID** | **Investigator ID (INVID)** | **Investigator name (INVNAME)** | **Subject ID (SUBID)** | **Unique subject ID (USUBID)** | **Age (yrs)** |  | **AE start date** | **AE end date** | **Verbatim term** |
| --- | --- | --- | --- | --- | --- | --- | --- | --- | --- |
| **00123** | **279344** | **Dr Smith** | **5** | **TJF4392.005** | **57** |  | **29DEC2010** | **27JAN2011** | **Headache** |
| **00123** | **279344** | **Dr Smith** | **2** | **TJF4392.002** | **72** |  | **10JAN2011** | **06APR2011** | **Nausea** |
| **00123** | **279344** | **Dr Smith** | **1** | **TJF4392.001** | **91** |  | **25MAR2011** | **12AUG2011** | **Cold** |
| **00123** | **279344** | **Dr Smith** | **66** | **TJF4392.066** | **89** |  | **28MAR2011** | **31MAR2011** | **Cold** |
| **00123** | **279344** | **Dr Smith** | **8** | **TJF4392.008** | **94** |  | **01MAR2011** | **15MAY2011** | **Flu** |
| **05678** | **333721** | **Dr Jones** | **19** | **TJF4392.019** | **85** |  | **14OCT2010** | **20OCT2011** | **Cold** |
| **05678** | **333721** | **Dr Jones** | **4** | **TJF4392.004** | **53** |  | **24MAY2011** | **.** | **Headache** |
| **05678** | **333721** | **Dr Jones** | **23** | **TJF4392.002** | **76** |  | **01MAR2011** | **15MAR2011** | **Pain** |

| **New centre ID. Merged as < 10 patients** | | | **Remove INVID** | **Drop INVNAME from dataset** | | **New SUBID** | | | **New USUBID** | **Remove ages above 89** | | | **Create age category** | **Calculate study day** | | | **Calculate study day** | **Remove** | |
| --- | --- | --- | --- | --- | --- | --- | --- | --- | --- | --- | --- | --- | --- | --- | --- | --- | --- | --- | --- |
| **Centre ID** | **Investigator ID (INVID)** | | |  | | **Subject ID (SUBID)** | **Unique subject ID (USUBID)** | | | **Age (yrs)** | **Age Category** | | | **AE start date** | **AE end date** | | | **Verbatim term** | |
| **22265** |  | | |  |  | **8754** | **TJF4392.8754** | | | **57** | **<=89** | | | **20** | **49** | | |  | |
| **22265** |  | | |  |  | **5681** | **TJF4392.5681** | | | **72** | **<=89** | | | **15** | **192** | | |  | |
| **22265** |  | | |  |  | **1475** | **TJF4392.1475** | | | **.** | **>89** | | | **322** | **462** | | |  | |
| **22265** |  | | |  |  | **6589** | **TJF4392.6589** | | | **89** | **<=89** | | | **17** | **20** | | |  | |
| **22265** |  | | |  |  | **3562** | **TJF4392.3562** | | | **94** | **>89** | | | **23** | **98** | | |  | |
| **22265** |  | | |  |  | **1457** | **TJF4392.1457** | | | **.** | **<=89** | | | **2** | **312** | | |  | |
| **22265** |  | | |  |  | **2214** | **TJF4392.2214** | | | **53** | **<=89** | | | **4** | **.** | | |  | |
| **22265** |  | | |  |  | **2236** | **TJF4392.2236** | | | **76** | **<=89** | | | **15** | **29** | | |  | |

# Appendix 4: Data Sharing Workshop Attendees

This guidance document was advised by an expert committee who met in November 2014 to discuss and provide feedback on an initial draft of the document.

Attendees:

| **Name** | **Organisation** |
| --- | --- |
| Sue Bell | Leeds Clinical Trials Research Unit |
| Jesse Berlin | Johnson & Johnson |
| Claire Daffern | Warwick Clinical Trial Unit |
| Rob Frost | GlaxoSmithKline |
| Jamie Garner | Keele Clinical Trial Unit |
| Will Greenacre | The Wellcome Trust |
| Sally Hollis | AstraZeneca |
| Carolyn Hopkins | Liverpool Clinical Trials Research Centre |
| Nazir Lone | University of Edinburgh |
| Gordon Murray | University of Edinburgh |
| Maike Rentel | The Wellcome Trust/Technopolis |
| Catrin Tudur Smith | North West Hub for Trials Methodology Research |
| Lesley Stewart | University of York |
| Matt Sydes | MRC Clinical Trial Unit |
| Liz Tremain | National Institute of Health Research |
| Peter Varnai | The Wellcome Trust/Technopolis |
| Kerry Woolfall | University of Liverpool |

The following people also provided feedback on the initial draft of this document but were unable to attend the workshop:

| **Name** | **Organisation** |
| --- | --- |
| Richard Riley | University of Keele |
| Haleema Shakur | London School of Hygiene and Tropical Medicine |
| Paula Williamson | North West Hub for Trials Methodology Research |

1. Hrynaszkiewicz I, Norton ML, *et al.* Preparing raw clinical data for publication: guidance for journal editors, authors, and peer reviewers. *BMJ* 2010; **340**: c181. [↑](#footnote-ref-2)
2. De-identification of Clinical Trials Data Demystified. *Jack Shostak, Duke Clinical Research Institute (DCRI), Durham, NC* [*http://www.lexjansen.com/pharmasug/2006/publichealthresearch/pr02.pdf*](http://www.lexjansen.com/pharmasug/2006/publichealthresearch/pr02.pdf) [↑](#footnote-ref-3)
